# 6,7,8,14,15,16-Hexaphenyldibenzo[*c*,*gh*]naphtho[3,2,1,8-*pqra*]tetraphene-5,13-dione dichloromethane monosolvate

**DOI:** 10.1107/S1600536812013621

**Published:** 2012-04-04

**Authors:** Shuhong Li, Limin Chen, Caihong Xu

**Affiliations:** a Department of Chemistry, School of Science, Beijing Technology and Business University, Beijing 100048, People’s Republic of China; b Institute of Chemistry, Chinese Academy of Sciences, Beijing 100190, People’s Republic of China

## Abstract

The main mol­ecule of the title compound, C_66_H_38_O_2_·CH_2_Cl_2_, is centrosymmetric, the asymmetric unit is composed of two half-mol­ecules, located on inversion centers, and a mol­ecule of dichloro­methane. The large π-conjugated fused polycyclic system including eight six-membered rings is nearly planar, with r.m.s. deviations of 0.2114 and 0.2081 Å in the two independent mol­ecules.

## Related literature

For investigations of polycyclic aromatic acenes, see Bendikov *et al.* (2004 ▶); Anthony (2006 ▶); Pascal (2006 ▶).
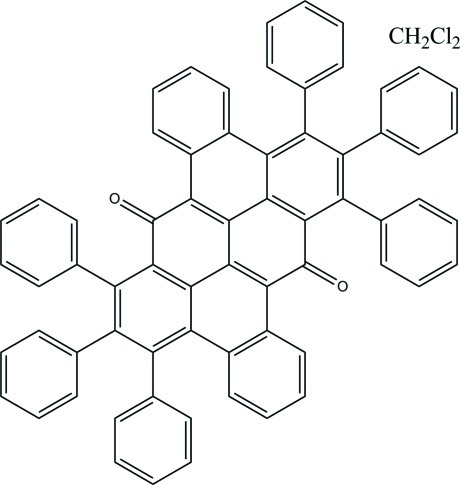


## Experimental

### 

#### Crystal data

C_66_H_38_O_2_·CH_2_Cl_2_
*M* *_r_* = 947.89
Triclinic, 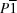

*a* = 12.587 (3) Å
*b* = 13.466 (3) Å
*c* = 15.872 (3) Å
α = 91.82 (3)°
β = 107.75 (3)°
γ = 112.20 (3)°
*V* = 2339.0 (12) Å^3^
*Z* = 2
Mo *K*α radiation
μ = 0.19 mm^−1^
*T* = 173 K
0.28 × 0.22 × 0.10 mm

#### Data collection

Rigaku R-AXIS RAPID IP area-detector diffractometer
Absorption correction: multi-scan (*ABSCOR*; Higashi, 1995 ▶) *T*_min_ = 0.949, *T*_max_ = 0.981
15194 measured reflections
8220 independent reflections
6068 reflections with *I* > 2σ(*I*)
*R*_int_ = 0.045

#### Refinement

*R*[*F* ^2^ > 2σ(*F* ^2^)] = 0.087
*wR*(*F* ^2^) = 0.148
*S* = 1.26
8220 reflections
640 parameters
H-atom parameters constrained
Δρ_max_ = 0.32 e Å^−3^
Δρ_min_ = −0.26 e Å^−3^

### 

Data collection: *RAPID-AUTO* (Rigaku, 2001 ▶); cell refinement: *RAPID-AUTO*; data reduction: *RAPID-AUTO*; program(s) used to solve structure: *SHELXS97* (Sheldrick, 2008 ▶); program(s) used to refine structure: *SHELXL97* (Sheldrick, 2008 ▶); molecular graphics: *SHELXTL* (Sheldrick, 2008 ▶); software used to prepare material for publication: *SHELXL97*.

## Supplementary Material

Crystal structure: contains datablock(s) I, global. DOI: 10.1107/S1600536812013621/aa2047sup1.cif

Structure factors: contains datablock(s) I. DOI: 10.1107/S1600536812013621/aa2047Isup2.hkl

Supplementary material file. DOI: 10.1107/S1600536812013621/aa2047Isup3.cml

Additional supplementary materials:  crystallographic information; 3D view; checkCIF report

## Figures and Tables

**Fig. 1.**
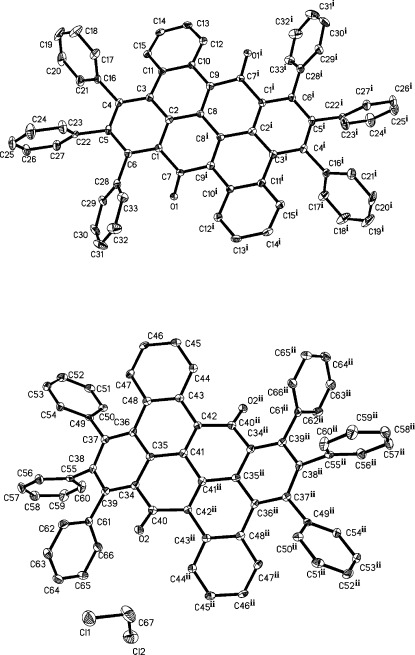
The structure of the title compound showing atom labelling and 30% probability displacement ellipsoids for non-H atoms. Symmetry codes: (i) x, -y, -z; (ii) 1 - x, 1 - y, -z.

## supplementary crystallographic information

### Comment

Polycyclic aromatic acenes have received considerable attention in the past few decades (Bendikov *et al.*, 2004; Anthony, 2006; Pascal, 2006) due to their potential application for construction organic electronic devices. On the investigation of partially fluorinated polycyclic aromatic compounds, the title molecule was separated as a byproduct in a designed route. The molecular structure of the title compound is shown in Fig. 1. The main molecule of the title compound is centrosymmetric, the asymmetric unit is built from two halves of the molecule arranged around inversion centers and a molecule of dichloromethane. The large π-conjugated fused polycyclic system including eight six-membered rings is nearly planar. The related RMS deviations are 0.2114 Å (for the plane formed by C1 to C15, and C1^i^ to C15^i^) and 0.2081 Å (for the plane formed by C34 to C48, and C34^ii^ to C48^ii^). Symmetry codes correspond to Fig. 1. There are 12 phenyl groups linked to both π-conjugated fused polycyclics and 6 of them are unique with dihedral angles of 66.549 (130)°, 86.579 (122)°, 65.995 (102)°, 60.652 (87)°, 87.325 (119)°, and 76.421 (113)°.

### Experimental

A mixture of granular lithium (18.2 mg, 2.6 mmol) and naphthalene (336.3 mg, 2.6 mmol) in THF was stirred at room temperature (rt) for 4 h. To the resulting solution of lithium naphthalenide, a solution of diphenylacetylene (311.6 mg, 1.75 mmol) in THF (4 mL) was added at room temperature. After stirring for 20 min, perfluoronaphthalene (123 mg, 0.45 mmol) in THF (5 ml) was added to the reaction mixture at room temperature. The reaction mixture was stirred for 1 h and then quenched with a saturated aqueous solution of NH_4_Cl. The mixture was extracted with ethyl ether. The organic layer was washed with brine, dried over MgSO_4_, filtered, and concentrated under reduced pressure. The resulting mixture was gradually passed through a silica gel column with different ratio of petroleum ether/ethyl acetate mixture as an eluent, followed by further purification by recrystallization (CH_2_Cl_2_/acetone) to give small amount as a yellow-brown crystal.

### Refinement

H atoms were located in a difference map, placed geometrically and refined using a riding model with C—H distances 0.99 Å (dichloromethane) and 0.95 Å (aromatic), and with *U*_iso_(H) = 1.2 *U*_eq_(C).

### Crystal data

**Table:**

C_66_H_38_O_2_·CH_2_Cl_2_	*Z* = 2
*M**_r_* = 947.89	*F*(000) = 984
Triclinic, *P*1	*D*_x_ = 1.346 Mg m^−^^3^
Hall symbol: -P 1	Mo *K*α radiation, λ = 0.71073 Å
*a* = 12.587 (3) Å	Cell parameters from 17659 reflections
*b* = 13.466 (3) Å	θ = 1.8–27.5°
*c* = 15.872 (3) Å	µ = 0.19 mm^−^^1^
α = 91.82 (3)°	*T* = 173 K
β = 107.75 (3)°	Plate, yellow
γ = 112.20 (3)°	0.28 × 0.22 × 0.10 mm
*V* = 2339.0 (12) Å^3^	

### Data collection

**Table:**

Rigaku R-AXIS RAPID IP area-detector diffractometer	8220 independent reflections
Radiation source: rotating anode	6068 reflections with *I* > 2σ(*I*)
Graphite monochromator	*R*_int_ = 0.045
ω scans at fixed χ = 45°	θ_max_ = 25.0°, θ_min_ = 1.9°
Absorption correction: multi-scan (*ABSCOR*; Higashi, 1995)	*h* = −14→14
*T*_min_ = 0.949, *T*_max_ = 0.981	*k* = −16→16
15194 measured reflections	*l* = −18→18

### Refinement

**Table:**

Refinement on *F*^2^	Primary atom site location: structure-invariant direct methods
Least-squares matrix: full	Secondary atom site location: difference Fourier map
*R*[*F*^2^ > 2σ(*F*^2^)] = 0.087	Hydrogen site location: inferred from neighbouring sites
*wR*(*F*^2^) = 0.148	H-atom parameters constrained
*S* = 1.26	*w* = 1/[σ^2^(*F*_o_^2^) + (0.0507*P*)^2^ + 0.1946*P*] where *P* = (*F*_o_^2^ + 2*F*_c_^2^)/3
8220 reflections	(Δ/σ)_max_ < 0.001
640 parameters	Δρ_max_ = 0.32 e Å^−^^3^
0 restraints	Δρ_min_ = −0.26 e Å^−^^3^

### Special details

**Table:**

Geometry. All e.s.d.'s (except the e.s.d. in the dihedral angle between two l.s. planes) are estimated using the full covariance matrix. The cell e.s.d.'s are taken into account individually in the estimation of e.s.d.'s in distances, angles and torsion angles; correlations between e.s.d.'s in cell parameters are only used when they are defined by crystal symmetry. An approximate (isotropic) treatment of cell e.s.d.'s is used for estimating e.s.d.'s involving l.s. planes.
Refinement. Refinement of *F*^2^ against ALL reflections. The weighted *R*-factor *wR* and goodness of fit *S* are based on *F*^2^, conventional *R*-factors *R* are based on *F*, with *F* set to zero for negative *F*^2^. The threshold expression of *F*^2^ > σ(*F*^2^) is used only for calculating *R*-factors(gt) *etc*. and is not relevant to the choice of reflections for refinement. *R*-factors based on *F*^2^ are statistically about twice as large as those based on *F*, and *R*- factors based on ALL data will be even larger.

### Fractional atomic coordinates and isotropic or equivalent isotropic displacement parameters (Å^2^)

**Table:**

	*x*	*y*	*z*	*U*_iso_*/*U*_eq_	
O1	0.6200 (2)	−0.19241 (17)	−0.05996 (14)	0.0241 (5)	
C1	0.6222 (3)	−0.1085 (2)	0.0740 (2)	0.0183 (7)	
C2	0.5476 (3)	−0.0681 (2)	0.1014 (2)	0.0200 (7)	
C3	0.5345 (3)	−0.0813 (2)	0.1860 (2)	0.0206 (7)	
C4	0.6146 (3)	−0.1179 (2)	0.2480 (2)	0.0219 (8)	
C5	0.6990 (3)	−0.1452 (2)	0.2232 (2)	0.0220 (8)	
C6	0.6993 (3)	−0.1455 (2)	0.1349 (2)	0.0206 (7)	
C7	0.6099 (3)	−0.1176 (2)	−0.0222 (2)	0.0191 (7)	
C8	0.4887 (3)	−0.0099 (2)	0.0422 (2)	0.0180 (7)	
C9	0.4234 (3)	0.0361 (2)	0.0713 (2)	0.0203 (7)	
C10	0.3867 (3)	0.0053 (2)	0.1482 (2)	0.0199 (7)	
C11	0.4372 (3)	−0.0582 (2)	0.2029 (2)	0.0216 (8)	
C12	0.2907 (3)	0.0256 (3)	0.1631 (2)	0.0259 (8)	
H12	0.2598	0.0714	0.1288	0.031*	
C13	0.2416 (3)	−0.0196 (3)	0.2258 (2)	0.0294 (9)	
H13	0.1778	−0.0046	0.2352	0.035*	
C14	0.2854 (3)	−0.0874 (3)	0.2753 (2)	0.0275 (8)	
H14	0.2483	−0.1219	0.3165	0.033*	
C15	0.3814 (3)	−0.1052 (3)	0.2657 (2)	0.0243 (8)	
H15	0.4115	−0.1502	0.3019	0.029*	
C16	0.6169 (3)	−0.1202 (3)	0.3423 (2)	0.0248 (8)	
C17	0.6572 (3)	−0.0237 (3)	0.4006 (2)	0.0345 (9)	
H17	0.6861	0.0438	0.3804	0.041*	
C18	0.6560 (4)	−0.0243 (4)	0.4865 (3)	0.0523 (12)	
H18	0.6835	0.0425	0.5248	0.063*	
C19	0.6154 (4)	−0.1207 (4)	0.5176 (3)	0.0574 (13)	
H19	0.6138	−0.1208	0.5771	0.069*	
C20	0.5767 (4)	−0.2176 (4)	0.4616 (3)	0.0518 (13)	
H20	0.5493	−0.2845	0.4831	0.062*	
C21	0.5774 (3)	−0.2184 (3)	0.3744 (3)	0.0358 (9)	
H21	0.5512	−0.2854	0.3366	0.043*	
C22	0.7968 (3)	−0.1662 (3)	0.2930 (2)	0.0216 (8)	
C23	0.9030 (3)	−0.0792 (3)	0.3428 (3)	0.0390 (10)	
H23	0.9121	−0.0076	0.3334	0.047*	
C24	0.9962 (4)	−0.0943 (4)	0.4061 (3)	0.0524 (12)	
H24	1.0686	−0.0332	0.4399	0.063*	
C25	0.9851 (4)	−0.1976 (4)	0.4208 (3)	0.0483 (11)	
H25	1.0493	−0.2082	0.4643	0.058*	
C26	0.8793 (4)	−0.2854 (3)	0.3713 (3)	0.0408 (10)	
H26	0.8711	−0.3569	0.3805	0.049*	
C27	0.7847 (3)	−0.2707 (3)	0.3082 (2)	0.0319 (9)	
H27	0.7116	−0.3317	0.2754	0.038*	
C28	0.7875 (3)	−0.1761 (3)	0.1067 (2)	0.0236 (8)	
C29	0.7741 (3)	−0.2831 (3)	0.0977 (2)	0.0296 (9)	
H29	0.7135	−0.3363	0.1153	0.036*	
C30	0.8479 (3)	−0.3131 (3)	0.0633 (3)	0.0397 (10)	
H30	0.8362	−0.3872	0.0557	0.048*	
C31	0.9380 (3)	−0.2368 (3)	0.0399 (3)	0.0477 (12)	
H31	0.9882	−0.2580	0.0159	0.057*	
C32	0.9554 (3)	−0.1291 (4)	0.0516 (3)	0.0475 (11)	
H32	1.0187	−0.0756	0.0367	0.057*	
C33	0.8802 (3)	−0.0991 (3)	0.0851 (2)	0.0327 (9)	
H33	0.8926	−0.0248	0.0932	0.039*	
O2	0.6914 (2)	0.44986 (18)	0.20654 (15)	0.0248 (5)	
C34	0.5015 (3)	0.4662 (2)	0.1592 (2)	0.0193 (7)	
C35	0.4428 (3)	0.5163 (2)	0.0961 (2)	0.0189 (7)	
C36	0.3578 (3)	0.5520 (2)	0.1143 (2)	0.0187 (7)	
C37	0.3122 (3)	0.5103 (2)	0.1830 (2)	0.0199 (7)	
C38	0.3599 (3)	0.4466 (3)	0.2371 (2)	0.0215 (8)	
C39	0.4658 (3)	0.4350 (2)	0.2322 (2)	0.0194 (7)	
C40	0.6024 (3)	0.4448 (2)	0.1444 (2)	0.0200 (7)	
C41	0.4642 (3)	0.5269 (2)	0.0116 (2)	0.0191 (7)	
C42	0.4053 (3)	0.5751 (2)	−0.0501 (2)	0.0181 (7)	
C43	0.3424 (3)	0.6348 (2)	−0.0249 (2)	0.0189 (7)	
C44	0.3096 (3)	0.7072 (2)	−0.0787 (2)	0.0204 (7)	
H44	0.3259	0.7142	−0.1333	0.025*	
C45	0.2547 (3)	0.7672 (3)	−0.0534 (2)	0.0252 (8)	
H45	0.2316	0.8146	−0.0907	0.030*	
C46	0.2327 (3)	0.7588 (3)	0.0272 (2)	0.0230 (8)	
H46	0.1945	0.8006	0.0446	0.028*	
C47	0.2655 (3)	0.6906 (3)	0.0823 (2)	0.0229 (8)	
H47	0.2515	0.6877	0.1379	0.027*	
C48	0.3195 (3)	0.6251 (2)	0.0577 (2)	0.0195 (7)	
C49	0.2058 (3)	0.5252 (2)	0.1962 (2)	0.0207 (7)	
C50	0.0913 (3)	0.4819 (3)	0.1298 (2)	0.0235 (8)	
H50	0.0789	0.4395	0.0759	0.028*	
C51	−0.0042 (3)	0.4997 (3)	0.1411 (2)	0.0291 (9)	
H51	−0.0819	0.4700	0.0949	0.035*	
C52	0.0118 (3)	0.5608 (3)	0.2194 (3)	0.0340 (9)	
H52	−0.0545	0.5735	0.2266	0.041*	
C53	0.1241 (3)	0.6031 (3)	0.2868 (2)	0.0318 (9)	
H53	0.1351	0.6443	0.3409	0.038*	
C54	0.2213 (3)	0.5854 (3)	0.2759 (2)	0.0248 (8)	
H54	0.2985	0.6144	0.3226	0.030*	
C55	0.2890 (3)	0.3757 (3)	0.2897 (2)	0.0222 (8)	
C56	0.3100 (3)	0.4014 (3)	0.3796 (2)	0.0288 (8)	
H56	0.3741	0.4680	0.4135	0.035*	
C57	0.2376 (3)	0.3300 (3)	0.4208 (3)	0.0350 (9)	
H57	0.2517	0.3488	0.4826	0.042*	
C58	0.1460 (3)	0.2327 (3)	0.3734 (3)	0.0349 (9)	
H58	0.0967	0.1844	0.4021	0.042*	
C59	0.1260 (4)	0.2055 (3)	0.2845 (3)	0.0407 (10)	
H59	0.0638	0.1376	0.2515	0.049*	
C60	0.1965 (3)	0.2769 (3)	0.2428 (3)	0.0363 (10)	
H60	0.1813	0.2580	0.1808	0.044*	
C61	0.5275 (3)	0.3821 (3)	0.3003 (2)	0.0197 (7)	
C62	0.6089 (3)	0.4428 (3)	0.3831 (2)	0.0313 (9)	
H62	0.6216	0.5165	0.3969	0.038*	
C63	0.6721 (3)	0.3976 (3)	0.4458 (2)	0.0348 (9)	
H63	0.7288	0.4404	0.5019	0.042*	
C64	0.6521 (3)	0.2904 (3)	0.4265 (3)	0.0346 (9)	
H64	0.6949	0.2588	0.4693	0.041*	
C65	0.5699 (3)	0.2286 (3)	0.3448 (3)	0.0342 (9)	
H65	0.5556	0.1543	0.3317	0.041*	
C66	0.5088 (3)	0.2749 (3)	0.2823 (2)	0.0283 (8)	
H66	0.4529	0.2322	0.2259	0.034*	
Cl1	0.96908 (11)	0.39486 (10)	0.41839 (8)	0.0611 (3)	
Cl2	0.82255 (11)	0.19108 (9)	0.29124 (8)	0.0569 (3)	
C67	0.8978 (5)	0.3331 (3)	0.3061 (3)	0.0652 (14)	
H67A	0.9600	0.3517	0.2764	0.078*	
H67B	0.8379	0.3630	0.2762	0.078*	

### Atomic displacement parameters (Å^2^)

**Table:**

	*U*^11^	*U*^22^	*U*^33^	*U*^12^	*U*^13^	*U*^23^
O1	0.0308 (14)	0.0261 (13)	0.0199 (14)	0.0173 (11)	0.0076 (11)	0.0015 (10)
C1	0.0167 (17)	0.0162 (17)	0.021 (2)	0.0048 (14)	0.0072 (14)	0.0049 (13)
C2	0.0170 (17)	0.0172 (17)	0.020 (2)	0.0022 (14)	0.0048 (14)	−0.0001 (13)
C3	0.0198 (17)	0.0184 (17)	0.019 (2)	0.0048 (14)	0.0046 (15)	0.0015 (13)
C4	0.0248 (18)	0.0196 (18)	0.0161 (19)	0.0051 (15)	0.0050 (15)	0.0040 (13)
C5	0.0214 (18)	0.0169 (17)	0.022 (2)	0.0052 (14)	0.0024 (15)	0.0066 (14)
C6	0.0211 (18)	0.0163 (17)	0.022 (2)	0.0062 (14)	0.0056 (15)	0.0035 (13)
C7	0.0180 (17)	0.0196 (18)	0.020 (2)	0.0079 (14)	0.0068 (15)	0.0002 (14)
C8	0.0177 (17)	0.0150 (16)	0.0179 (19)	0.0043 (14)	0.0049 (14)	−0.0003 (13)
C9	0.0167 (17)	0.0174 (17)	0.021 (2)	0.0031 (14)	0.0037 (14)	−0.0009 (13)
C10	0.0199 (17)	0.0196 (17)	0.018 (2)	0.0068 (14)	0.0059 (15)	0.0021 (13)
C11	0.0238 (18)	0.0199 (18)	0.017 (2)	0.0068 (15)	0.0048 (15)	−0.0008 (13)
C12	0.031 (2)	0.0257 (19)	0.024 (2)	0.0142 (16)	0.0096 (17)	0.0054 (15)
C13	0.028 (2)	0.038 (2)	0.029 (2)	0.0170 (17)	0.0152 (17)	0.0022 (17)
C14	0.030 (2)	0.030 (2)	0.023 (2)	0.0082 (16)	0.0163 (17)	0.0030 (15)
C15	0.028 (2)	0.0250 (19)	0.021 (2)	0.0097 (16)	0.0105 (16)	0.0045 (14)
C16	0.0206 (18)	0.036 (2)	0.020 (2)	0.0150 (16)	0.0061 (15)	0.0091 (16)
C17	0.027 (2)	0.045 (2)	0.023 (2)	0.0074 (17)	0.0081 (17)	0.0034 (17)
C18	0.048 (3)	0.070 (3)	0.024 (3)	0.008 (2)	0.013 (2)	−0.004 (2)
C19	0.047 (3)	0.102 (4)	0.017 (3)	0.024 (3)	0.011 (2)	0.012 (3)
C20	0.046 (3)	0.079 (3)	0.049 (3)	0.031 (3)	0.031 (2)	0.051 (3)
C21	0.034 (2)	0.042 (2)	0.037 (3)	0.0186 (18)	0.0145 (19)	0.0189 (18)
C22	0.0283 (19)	0.0295 (19)	0.0126 (19)	0.0169 (16)	0.0079 (15)	0.0060 (14)
C23	0.032 (2)	0.031 (2)	0.043 (3)	0.0125 (18)	−0.0022 (19)	0.0068 (17)
C24	0.035 (2)	0.050 (3)	0.050 (3)	0.013 (2)	−0.009 (2)	0.005 (2)
C25	0.041 (3)	0.067 (3)	0.038 (3)	0.033 (2)	−0.001 (2)	0.017 (2)
C26	0.053 (3)	0.047 (3)	0.038 (3)	0.035 (2)	0.016 (2)	0.022 (2)
C27	0.034 (2)	0.032 (2)	0.029 (2)	0.0159 (17)	0.0066 (18)	0.0093 (16)
C28	0.0183 (18)	0.0246 (19)	0.022 (2)	0.0076 (15)	0.0001 (15)	0.0021 (14)
C29	0.029 (2)	0.028 (2)	0.034 (2)	0.0161 (17)	0.0066 (17)	0.0065 (16)
C30	0.036 (2)	0.037 (2)	0.048 (3)	0.026 (2)	0.003 (2)	−0.0032 (19)
C31	0.022 (2)	0.056 (3)	0.063 (3)	0.017 (2)	0.013 (2)	−0.013 (2)
C32	0.026 (2)	0.054 (3)	0.055 (3)	0.005 (2)	0.019 (2)	−0.005 (2)
C33	0.028 (2)	0.030 (2)	0.037 (2)	0.0093 (17)	0.0093 (17)	−0.0011 (16)
O2	0.0189 (12)	0.0347 (14)	0.0218 (14)	0.0145 (11)	0.0037 (11)	0.0064 (10)
C34	0.0145 (17)	0.0206 (18)	0.021 (2)	0.0059 (14)	0.0062 (15)	0.0003 (14)
C35	0.0134 (16)	0.0191 (17)	0.021 (2)	0.0059 (14)	0.0028 (14)	0.0014 (13)
C36	0.0142 (16)	0.0193 (17)	0.0192 (19)	0.0043 (14)	0.0050 (14)	−0.0030 (13)
C37	0.0156 (16)	0.0194 (17)	0.021 (2)	0.0058 (14)	0.0038 (14)	−0.0002 (13)
C38	0.0226 (18)	0.0225 (18)	0.019 (2)	0.0073 (15)	0.0087 (15)	−0.0006 (14)
C39	0.0148 (17)	0.0199 (17)	0.021 (2)	0.0061 (14)	0.0042 (14)	−0.0012 (13)
C40	0.0166 (17)	0.0171 (17)	0.023 (2)	0.0038 (14)	0.0069 (15)	0.0038 (13)
C41	0.0127 (16)	0.0187 (17)	0.022 (2)	0.0026 (13)	0.0065 (14)	0.0020 (14)
C42	0.0169 (17)	0.0160 (17)	0.022 (2)	0.0060 (14)	0.0079 (15)	0.0031 (13)
C43	0.0119 (16)	0.0161 (17)	0.027 (2)	0.0030 (13)	0.0075 (15)	0.0047 (14)
C44	0.0141 (16)	0.0227 (18)	0.022 (2)	0.0057 (14)	0.0056 (14)	0.0028 (14)
C45	0.0221 (18)	0.0192 (18)	0.035 (2)	0.0087 (15)	0.0095 (16)	0.0062 (15)
C46	0.0178 (17)	0.0215 (18)	0.032 (2)	0.0112 (14)	0.0075 (16)	0.0005 (15)
C47	0.0192 (17)	0.0243 (18)	0.027 (2)	0.0087 (15)	0.0107 (15)	0.0009 (15)
C48	0.0091 (15)	0.0187 (17)	0.026 (2)	0.0021 (13)	0.0043 (14)	0.0007 (14)
C49	0.0246 (18)	0.0200 (17)	0.026 (2)	0.0127 (15)	0.0148 (16)	0.0130 (14)
C50	0.0222 (18)	0.0203 (18)	0.028 (2)	0.0098 (15)	0.0072 (16)	0.0044 (14)
C51	0.0170 (18)	0.031 (2)	0.040 (2)	0.0107 (16)	0.0081 (17)	0.0116 (17)
C52	0.030 (2)	0.041 (2)	0.050 (3)	0.0233 (18)	0.027 (2)	0.0200 (19)
C53	0.042 (2)	0.039 (2)	0.031 (2)	0.0253 (19)	0.0235 (19)	0.0115 (17)
C54	0.0259 (19)	0.030 (2)	0.024 (2)	0.0137 (16)	0.0124 (16)	0.0069 (15)
C55	0.0225 (18)	0.0251 (19)	0.024 (2)	0.0155 (15)	0.0074 (15)	0.0069 (14)
C56	0.030 (2)	0.028 (2)	0.024 (2)	0.0093 (16)	0.0073 (17)	0.0035 (15)
C57	0.042 (2)	0.044 (2)	0.024 (2)	0.018 (2)	0.0165 (19)	0.0132 (18)
C58	0.038 (2)	0.035 (2)	0.041 (3)	0.0143 (19)	0.024 (2)	0.0163 (18)
C59	0.040 (2)	0.033 (2)	0.044 (3)	0.0034 (18)	0.021 (2)	0.0007 (18)
C60	0.038 (2)	0.037 (2)	0.030 (2)	0.0068 (18)	0.0180 (19)	−0.0005 (17)
C61	0.0132 (16)	0.0283 (19)	0.021 (2)	0.0086 (14)	0.0097 (14)	0.0070 (14)
C62	0.033 (2)	0.029 (2)	0.030 (2)	0.0163 (17)	0.0044 (17)	−0.0010 (16)
C63	0.034 (2)	0.046 (2)	0.021 (2)	0.0204 (19)	0.0005 (17)	0.0016 (17)
C64	0.035 (2)	0.045 (2)	0.032 (2)	0.0250 (19)	0.0118 (19)	0.0168 (18)
C65	0.044 (2)	0.029 (2)	0.038 (3)	0.0223 (18)	0.015 (2)	0.0117 (17)
C66	0.032 (2)	0.027 (2)	0.025 (2)	0.0122 (16)	0.0086 (17)	0.0056 (15)
Cl1	0.0554 (7)	0.0805 (9)	0.0456 (8)	0.0301 (7)	0.0117 (6)	0.0101 (6)
Cl2	0.0581 (7)	0.0486 (7)	0.0721 (9)	0.0212 (6)	0.0321 (6)	0.0263 (6)
C67	0.085 (4)	0.045 (3)	0.045 (3)	0.013 (3)	0.009 (3)	0.015 (2)

### Geometric parameters (Å, º)

**Table:**

O1—C7	1.223 (3)	C34—C35	1.405 (4)
C1—C6	1.395 (4)	C34—C40	1.485 (4)
C1—C2	1.409 (4)	C35—C36	1.424 (4)
C1—C7	1.483 (4)	C35—C41	1.447 (4)
C2—C3	1.411 (4)	C36—C37	1.418 (4)
C2—C8	1.451 (4)	C36—C48	1.470 (4)
C3—C4	1.425 (4)	C37—C38	1.399 (4)
C3—C11	1.467 (4)	C37—C49	1.499 (4)
C4—C5	1.400 (5)	C38—C39	1.424 (4)
C4—C16	1.490 (5)	C38—C55	1.504 (4)
C5—C6	1.403 (5)	C39—C61	1.495 (4)
C5—C22	1.508 (4)	C40—C42^ii^	1.480 (4)
C6—C28	1.497 (4)	C41—C42	1.374 (4)
C7—C9^i^	1.480 (4)	C41—C41^ii^	1.466 (6)
C8—C9	1.367 (4)	C42—C43	1.444 (4)
C8—C8^i^	1.465 (6)	C42—C40^ii^	1.480 (4)
C9—C10	1.452 (4)	C43—C44	1.411 (4)
C9—C7^i^	1.480 (4)	C43—C48	1.425 (4)
C10—C12	1.417 (4)	C44—C45	1.365 (4)
C10—C11	1.418 (4)	C44—H44	0.9500
C11—C15	1.421 (5)	C45—C46	1.388 (5)
C12—C13	1.368 (5)	C45—H45	0.9500
C12—H12	0.9500	C46—C47	1.376 (4)
C13—C14	1.384 (5)	C46—H46	0.9500
C13—H13	0.9500	C47—C48	1.410 (4)
C14—C15	1.368 (5)	C47—H47	0.9500
C14—H14	0.9500	C49—C50	1.389 (5)
C15—H15	0.9500	C49—C54	1.401 (4)
C16—C17	1.393 (5)	C50—C51	1.371 (5)
C16—C21	1.399 (5)	C50—H50	0.9500
C17—C18	1.367 (5)	C51—C52	1.386 (5)
C17—H17	0.9500	C51—H51	0.9500
C18—C19	1.372 (6)	C52—C53	1.378 (5)
C18—H18	0.9500	C52—H52	0.9500
C19—C20	1.384 (6)	C53—C54	1.389 (5)
C19—H19	0.9500	C53—H53	0.9500
C20—C21	1.386 (5)	C54—H54	0.9500
C20—H20	0.9500	C55—C56	1.377 (5)
C21—H21	0.9500	C55—C60	1.385 (5)
C22—C23	1.377 (5)	C56—C57	1.386 (5)
C22—C27	1.393 (4)	C56—H56	0.9500
C23—C24	1.379 (5)	C57—C58	1.371 (5)
C23—H23	0.9500	C57—H57	0.9500
C24—C25	1.379 (6)	C58—C59	1.367 (5)
C24—H24	0.9500	C58—H58	0.9500
C25—C26	1.379 (6)	C59—C60	1.380 (5)
C25—H25	0.9500	C59—H59	0.9500
C26—C27	1.387 (5)	C60—H60	0.9500
C26—H26	0.9500	C61—C66	1.377 (4)
C27—H27	0.9500	C61—C62	1.386 (5)
C28—C33	1.381 (5)	C62—C63	1.384 (5)
C28—C29	1.383 (4)	C62—H62	0.9500
C29—C30	1.381 (5)	C63—C64	1.376 (5)
C29—H29	0.9500	C63—H63	0.9500
C30—C31	1.371 (6)	C64—C65	1.380 (5)
C30—H30	0.9500	C64—H64	0.9500
C31—C32	1.381 (6)	C65—C66	1.380 (5)
C31—H31	0.9500	C65—H65	0.9500
C32—C33	1.386 (5)	C66—H66	0.9500
C32—H32	0.9500	Cl1—C67	1.744 (5)
C33—H33	0.9500	Cl2—C67	1.754 (4)
O2—C40	1.223 (4)	C67—H67A	0.9900
C34—C39	1.387 (4)	C67—H67B	0.9900
			
C6—C1—C2	120.6 (3)	C34—C35—C36	119.3 (3)
C6—C1—C7	121.2 (3)	C34—C35—C41	120.3 (3)
C2—C1—C7	118.1 (3)	C36—C35—C41	120.3 (3)
C1—C2—C3	120.1 (3)	C37—C36—C35	117.5 (3)
C1—C2—C8	118.6 (3)	C37—C36—C48	124.9 (3)
C3—C2—C8	121.2 (3)	C35—C36—C48	117.5 (3)
C2—C3—C4	118.0 (3)	C38—C37—C36	120.6 (3)
C2—C3—C11	117.3 (3)	C38—C37—C49	117.3 (3)
C4—C3—C11	124.7 (3)	C36—C37—C49	121.9 (3)
C5—C4—C3	120.5 (3)	C37—C38—C39	120.2 (3)
C5—C4—C16	118.5 (3)	C37—C38—C55	120.2 (3)
C3—C4—C16	120.9 (3)	C39—C38—C55	118.9 (3)
C4—C5—C6	120.4 (3)	C34—C39—C38	117.6 (3)
C4—C5—C22	120.1 (3)	C34—C39—C61	122.7 (3)
C6—C5—C22	119.3 (3)	C38—C39—C61	119.5 (3)
C1—C6—C5	119.3 (3)	O2—C40—C42^ii^	121.1 (3)
C1—C6—C28	119.3 (3)	O2—C40—C34	122.2 (3)
C5—C6—C28	121.3 (3)	C42^ii^—C40—C34	116.5 (3)
O1—C7—C9^i^	121.8 (3)	C42—C41—C35	119.8 (3)
O1—C7—C1	121.5 (3)	C42—C41—C41^ii^	120.8 (4)
C9^i^—C7—C1	116.6 (3)	C35—C41—C41^ii^	118.9 (4)
C9—C8—C2	118.7 (3)	C41—C42—C43	120.5 (3)
C9—C8—C8^i^	120.8 (4)	C41—C42—C40^ii^	118.4 (3)
C2—C8—C8^i^	120.1 (3)	C43—C42—C40^ii^	120.8 (3)
C8—C9—C10	120.9 (3)	C44—C43—C48	119.6 (3)
C8—C9—C7^i^	118.5 (3)	C44—C43—C42	120.7 (3)
C10—C9—C7^i^	120.6 (3)	C48—C43—C42	119.5 (3)
C12—C10—C11	119.2 (3)	C45—C44—C43	121.0 (3)
C12—C10—C9	121.2 (3)	C45—C44—H44	119.5
C11—C10—C9	119.1 (3)	C43—C44—H44	119.5
C10—C11—C15	117.2 (3)	C44—C45—C46	119.7 (3)
C10—C11—C3	119.0 (3)	C44—C45—H45	120.1
C15—C11—C3	123.4 (3)	C46—C45—H45	120.1
C13—C12—C10	121.3 (3)	C47—C46—C45	121.0 (3)
C13—C12—H12	119.3	C47—C46—H46	119.5
C10—C12—H12	119.3	C45—C46—H46	119.5
C12—C13—C14	119.6 (3)	C46—C47—C48	121.1 (3)
C12—C13—H13	120.2	C46—C47—H47	119.4
C14—C13—H13	120.2	C48—C47—H47	119.4
C15—C14—C13	120.8 (3)	C47—C48—C43	117.5 (3)
C15—C14—H14	119.6	C47—C48—C36	123.5 (3)
C13—C14—H14	119.6	C43—C48—C36	119.0 (3)
C14—C15—C11	121.6 (3)	C50—C49—C54	118.6 (3)
C14—C15—H15	119.2	C50—C49—C37	121.2 (3)
C11—C15—H15	119.2	C54—C49—C37	120.1 (3)
C17—C16—C21	118.2 (3)	C51—C50—C49	120.8 (3)
C17—C16—C4	120.5 (3)	C51—C50—H50	119.6
C21—C16—C4	121.3 (3)	C49—C50—H50	119.6
C18—C17—C16	121.3 (4)	C50—C51—C52	120.5 (3)
C18—C17—H17	119.3	C50—C51—H51	119.8
C16—C17—H17	119.3	C52—C51—H51	119.8
C17—C18—C19	120.6 (4)	C53—C52—C51	119.8 (3)
C17—C18—H18	119.7	C53—C52—H52	120.1
C19—C18—H18	119.7	C51—C52—H52	120.1
C18—C19—C20	119.3 (4)	C52—C53—C54	120.1 (3)
C18—C19—H19	120.3	C52—C53—H53	120.0
C20—C19—H19	120.3	C54—C53—H53	120.0
C19—C20—C21	120.8 (4)	C53—C54—C49	120.2 (3)
C19—C20—H20	119.6	C53—C54—H54	119.9
C21—C20—H20	119.6	C49—C54—H54	119.9
C20—C21—C16	119.8 (4)	C56—C55—C60	118.5 (3)
C20—C21—H21	120.1	C56—C55—C38	125.1 (3)
C16—C21—H21	120.1	C60—C55—C38	116.4 (3)
C23—C22—C27	118.6 (3)	C55—C56—C57	120.0 (3)
C23—C22—C5	118.9 (3)	C55—C56—H56	120.0
C27—C22—C5	122.4 (3)	C57—C56—H56	120.0
C22—C23—C24	121.1 (4)	C58—C57—C56	120.8 (4)
C22—C23—H23	119.4	C58—C57—H57	119.6
C24—C23—H23	119.4	C56—C57—H57	119.6
C25—C24—C23	120.4 (4)	C59—C58—C57	119.6 (3)
C25—C24—H24	119.8	C59—C58—H58	120.2
C23—C24—H24	119.8	C57—C58—H58	120.2
C24—C25—C26	119.0 (4)	C58—C59—C60	119.9 (4)
C24—C25—H25	120.5	C58—C59—H59	120.1
C26—C25—H25	120.5	C60—C59—H59	120.1
C25—C26—C27	120.9 (4)	C59—C60—C55	121.2 (4)
C25—C26—H26	119.6	C59—C60—H60	119.4
C27—C26—H26	119.6	C55—C60—H60	119.4
C26—C27—C22	120.0 (3)	C66—C61—C62	118.7 (3)
C26—C27—H27	120.0	C66—C61—C39	121.7 (3)
C22—C27—H27	120.0	C62—C61—C39	119.6 (3)
C33—C28—C29	118.8 (3)	C63—C62—C61	120.9 (3)
C33—C28—C6	120.7 (3)	C63—C62—H62	119.6
C29—C28—C6	120.5 (3)	C61—C62—H62	119.6
C30—C29—C28	120.5 (4)	C64—C63—C62	119.5 (3)
C30—C29—H29	119.8	C64—C63—H63	120.2
C28—C29—H29	119.8	C62—C63—H63	120.2
C31—C30—C29	120.5 (4)	C63—C64—C65	120.1 (3)
C31—C30—H30	119.7	C63—C64—H64	119.9
C29—C30—H30	119.7	C65—C64—H64	119.9
C30—C31—C32	119.6 (4)	C66—C65—C64	119.8 (3)
C30—C31—H31	120.2	C66—C65—H65	120.1
C32—C31—H31	120.2	C64—C65—H65	120.1
C31—C32—C33	119.9 (4)	C61—C66—C65	120.9 (3)
C31—C32—H32	120.0	C61—C66—H66	119.5
C33—C32—H32	120.0	C65—C66—H66	119.5
C28—C33—C32	120.6 (4)	Cl1—C67—Cl2	113.5 (2)
C28—C33—H33	119.7	Cl1—C67—H67A	108.9
C32—C33—H33	119.7	Cl2—C67—H67A	108.9
C39—C34—C35	121.9 (3)	Cl1—C67—H67B	108.9
C39—C34—C40	120.4 (3)	Cl2—C67—H67B	108.9
C35—C34—C40	117.7 (3)	H67A—C67—H67B	107.7
			
C6—C1—C2—C3	−10.6 (4)	C39—C34—C35—C36	10.2 (5)
C7—C1—C2—C3	165.1 (3)	C40—C34—C35—C36	−171.2 (3)
C6—C1—C2—C8	167.3 (3)	C39—C34—C35—C41	−166.8 (3)
C7—C1—C2—C8	−16.9 (4)	C40—C34—C35—C41	11.7 (4)
C1—C2—C3—C4	11.1 (4)	C34—C35—C36—C37	−16.3 (4)
C8—C2—C3—C4	−166.7 (3)	C41—C35—C36—C37	160.7 (3)
C1—C2—C3—C11	−167.7 (3)	C34—C35—C36—C48	166.5 (3)
C8—C2—C3—C11	14.4 (4)	C41—C35—C36—C48	−16.4 (4)
C2—C3—C4—C5	−3.3 (4)	C35—C36—C37—C38	7.1 (4)
C11—C3—C4—C5	175.4 (3)	C48—C36—C37—C38	−176.0 (3)
C2—C3—C4—C16	172.1 (3)	C35—C36—C37—C49	−168.2 (3)
C11—C3—C4—C16	−9.2 (5)	C48—C36—C37—C49	8.7 (5)
C3—C4—C5—C6	−5.2 (5)	C36—C37—C38—C39	8.6 (5)
C16—C4—C5—C6	179.2 (3)	C49—C37—C38—C39	−175.9 (3)
C3—C4—C5—C22	170.0 (3)	C36—C37—C38—C55	−161.5 (3)
C16—C4—C5—C22	−5.5 (4)	C49—C37—C38—C55	14.0 (4)
C2—C1—C6—C5	1.9 (5)	C35—C34—C39—C38	5.4 (4)
C7—C1—C6—C5	−173.7 (3)	C40—C34—C39—C38	−173.1 (3)
C2—C1—C6—C28	−173.1 (3)	C35—C34—C39—C61	−179.7 (3)
C7—C1—C6—C28	11.2 (4)	C40—C34—C39—C61	1.8 (5)
C4—C5—C6—C1	6.0 (5)	C37—C38—C39—C34	−14.9 (4)
C22—C5—C6—C1	−169.3 (3)	C55—C38—C39—C34	155.4 (3)
C4—C5—C6—C28	−179.1 (3)	C37—C38—C39—C61	170.1 (3)
C22—C5—C6—C28	5.6 (4)	C55—C38—C39—C61	−19.7 (4)
C6—C1—C7—O1	31.6 (4)	C39—C34—C40—O2	−35.6 (5)
C2—C1—C7—O1	−144.2 (3)	C35—C34—C40—O2	145.8 (3)
C6—C1—C7—C9^i^	−152.5 (3)	C39—C34—C40—C42^ii^	148.8 (3)
C2—C1—C7—C9^i^	31.7 (4)	C35—C34—C40—C42^ii^	−29.7 (4)
C1—C2—C8—C9	−175.3 (3)	C34—C35—C41—C42	179.4 (3)
C3—C2—C8—C9	2.6 (4)	C36—C35—C41—C42	2.3 (4)
C1—C2—C8—C8^i^	−2.6 (5)	C34—C35—C41—C41^ii^	7.3 (5)
C3—C2—C8—C8^i^	175.3 (3)	C36—C35—C41—C41^ii^	−169.7 (3)
C2—C8—C9—C10	−15.7 (4)	C35—C41—C42—C43	12.7 (4)
C8^i^—C8—C9—C10	171.6 (3)	C41^ii^—C41—C42—C43	−175.4 (3)
C2—C8—C9—C7^i^	164.8 (3)	C35—C41—C42—C40^ii^	−161.4 (3)
C8^i^—C8—C9—C7^i^	−7.9 (5)	C41^ii^—C41—C42—C40^ii^	10.5 (5)
C8—C9—C10—C12	−160.7 (3)	C41—C42—C43—C44	163.4 (3)
C7^i^—C9—C10—C12	18.8 (5)	C40^ii^—C42—C43—C44	−22.7 (4)
C8—C9—C10—C11	11.2 (4)	C41—C42—C43—C48	−13.0 (4)
C7^i^—C9—C10—C11	−169.3 (3)	C40^ii^—C42—C43—C48	160.9 (3)
C12—C10—C11—C15	4.7 (5)	C48—C43—C44—C45	−0.9 (4)
C9—C10—C11—C15	−167.4 (3)	C42—C43—C44—C45	−177.2 (3)
C12—C10—C11—C3	178.5 (3)	C43—C44—C45—C46	1.2 (5)
C9—C10—C11—C3	6.4 (4)	C44—C45—C46—C47	0.0 (5)
C2—C3—C11—C10	−18.7 (4)	C45—C46—C47—C48	−1.7 (5)
C4—C3—C11—C10	162.6 (3)	C46—C47—C48—C43	2.0 (4)
C2—C3—C11—C15	154.7 (3)	C46—C47—C48—C36	179.2 (3)
C4—C3—C11—C15	−24.0 (5)	C44—C43—C48—C47	−0.7 (4)
C11—C10—C12—C13	−3.5 (5)	C42—C43—C48—C47	175.7 (3)
C9—C10—C12—C13	168.5 (3)	C44—C43—C48—C36	−178.1 (3)
C10—C12—C13—C14	−0.6 (5)	C42—C43—C48—C36	−1.6 (4)
C12—C13—C14—C15	3.4 (5)	C37—C36—C48—C47	21.8 (5)
C13—C14—C15—C11	−2.1 (5)	C35—C36—C48—C47	−161.3 (3)
C10—C11—C15—C14	−2.0 (5)	C37—C36—C48—C43	−161.1 (3)
C3—C11—C15—C14	−175.5 (3)	C35—C36—C48—C43	15.9 (4)
C5—C4—C16—C17	109.5 (4)	C38—C37—C49—C50	−113.4 (4)
C3—C4—C16—C17	−66.0 (4)	C36—C37—C49—C50	62.0 (4)
C5—C4—C16—C21	−71.3 (4)	C38—C37—C49—C54	67.9 (4)
C3—C4—C16—C21	113.1 (4)	C36—C37—C49—C54	−116.6 (3)
C21—C16—C17—C18	−1.4 (5)	C54—C49—C50—C51	1.4 (5)
C4—C16—C17—C18	177.7 (3)	C37—C49—C50—C51	−177.3 (3)
C16—C17—C18—C19	0.3 (6)	C49—C50—C51—C52	−0.4 (5)
C17—C18—C19—C20	0.7 (7)	C50—C51—C52—C53	−0.6 (5)
C18—C19—C20—C21	−0.7 (7)	C51—C52—C53—C54	0.7 (5)
C19—C20—C21—C16	−0.4 (6)	C52—C53—C54—C49	0.3 (5)
C17—C16—C21—C20	1.4 (5)	C50—C49—C54—C53	−1.3 (5)
C4—C16—C21—C20	−177.7 (3)	C37—C49—C54—C53	177.4 (3)
C4—C5—C22—C23	−84.2 (4)	C37—C38—C55—C56	−100.0 (4)
C6—C5—C22—C23	91.1 (4)	C39—C38—C55—C56	89.7 (4)
C4—C5—C22—C27	96.8 (4)	C37—C38—C55—C60	80.5 (4)
C6—C5—C22—C27	−87.9 (4)	C39—C38—C55—C60	−89.8 (4)
C27—C22—C23—C24	0.4 (6)	C60—C55—C56—C57	−1.3 (5)
C5—C22—C23—C24	−178.6 (4)	C38—C55—C56—C57	179.2 (3)
C22—C23—C24—C25	0.2 (7)	C55—C56—C57—C58	1.1 (5)
C23—C24—C25—C26	−0.1 (7)	C56—C57—C58—C59	0.2 (6)
C24—C25—C26—C27	−0.6 (6)	C57—C58—C59—C60	−1.2 (6)
C25—C26—C27—C22	1.2 (6)	C58—C59—C60—C55	1.0 (6)
C23—C22—C27—C26	−1.1 (5)	C56—C55—C60—C59	0.3 (5)
C5—C22—C27—C26	177.9 (3)	C38—C55—C60—C59	179.8 (3)
C1—C6—C28—C33	63.8 (4)	C34—C39—C61—C66	−75.1 (4)
C5—C6—C28—C33	−111.2 (4)	C38—C39—C61—C66	99.7 (4)
C1—C6—C28—C29	−113.0 (4)	C34—C39—C61—C62	102.7 (4)
C5—C6—C28—C29	72.0 (4)	C38—C39—C61—C62	−82.5 (4)
C33—C28—C29—C30	−3.3 (5)	C66—C61—C62—C63	1.2 (5)
C6—C28—C29—C30	173.6 (3)	C39—C61—C62—C63	−176.7 (3)
C28—C29—C30—C31	1.9 (6)	C61—C62—C63—C64	−1.2 (6)
C29—C30—C31—C32	0.5 (6)	C62—C63—C64—C65	0.2 (6)
C30—C31—C32—C33	−1.3 (6)	C63—C64—C65—C66	0.7 (6)
C29—C28—C33—C32	2.5 (5)	C62—C61—C66—C65	−0.3 (5)
C6—C28—C33—C32	−174.4 (3)	C39—C61—C66—C65	177.6 (3)
C31—C32—C33—C28	−0.2 (6)	C64—C65—C66—C61	−0.6 (6)

Symmetry codes: (i) −*x*+1, −*y*, −*z*; (ii) −*x*+1, −*y*+1, −*z*.
